# The role of motivation in weight reduction programs for children and adolescents with overweight or obesity: a systematic review

**DOI:** 10.1007/s40519-025-01763-y

**Published:** 2025-07-09

**Authors:** Anne Herschbach, Annalena Wels, Pauline Dieckmann, Rebecca Erschens, Florian Junne, Stefan Ehehalt, Stephan Zipfel, Katrin Elisabeth Giel, Katrin Ziser

**Affiliations:** 1 Department of Psychosomatic Medicine and Psychotherapy, University Hospital Tuebingen, Tuebingen, Germany; 2 Department of Psychosomatic Medicine and Psychotherapy, Otto von Guericke University Magdeburg, Magdeburg, Germany; 3 Public Health Department of Stuttgart, Stuttgart, Germany; 4 German Center for Mental Health (DZPG), Tuebingen, Germany

**Keywords:** Obesity, Overweight, Children and adolescents, Motivation, Weight reduction

## Abstract

**Purpose:**

Obesity and overweight in children and adolescents are a growing concern. Treatments should be multidisciplinary, address the whole family and contain aspects of motivation. Motivation is a well-known impact factor in medical treatment but its role in the treatment of pediatric obesity is not clear. This systematic review aims to analyze the role of motivation by summarizing theoretical frameworks and synthesizing effects of weight reduction programs that assessed motivation as either outcome or predictor.

**Methods:**

This systematic review was conducted according to PRISMA guidelines. Four databases (PubMed, Web of Science, PsycINFO, Cochrane) were searched, findings were screened by two authors and assessed according to pre-determined criteria. Risk of bias was assessed with the Quality Assessment Tool for Quantitative Studies by the Effective Public Health Practice Project.

**Results:**

1899 studies were found, whereof 32 were included. The most commonly used theoretical frameworks were the Transtheoretical Model and Self-Determination Theory. In 18 of the included studies, motivation was assessed as outcome. In most of them, motivation increased towards the end of the weight reduction program. 15 studies used motivation to predict different outcome variables, however, with no conclusive evidence on the predictive power of motivation on different outcomes.

**Conclusion:**

Motivation can be enhanced over the course of a weight reduction program, mainly in pediatric patients. The predictive power of motivation on different outcomes is still not clear due to the heterogeneity of included studies. More research is needed to implement the current findings in future pediatric weight reduction programs.

*Level of evidence*: Level 1, systematic review.

## Introduction

Worldwide prevalence of obesity (OB) in children and adolescents is 6.9% in girls and 9.3% in boys [1]. Data show an increasing trend: the prevalence has nearly doubled since the 1990s [1]. In addition, the Covid-19 pandemic led to an increase in prevalence: In their systematic review, Anderson, et al. [2] found that in children worldwide, the OB prevalence increased by 2% and the pooled mean difference of BMI z-scores between before and during the pandemic was 0.13.

These trends are alarming because OB and overweight (OW) result in serious somatic and psychosocial secondary and concomitant diseases such as diabetes, joint complaints or mobbing, depression and lower quality of life [3–7]. The secondary diseases and especially psychosocial consequences can occur as early as in childhood and affect all areas of life. In addition, OB and OW in childhood is associated with a higher likelihood of OB and OW in adulthood [8] and premature mortality [9]. Treatment options for children and adolescents are therefore strongly needed and should start as early as possible to avoid the development of secondary and concomitant diseases.

The first treatment of choice for OB and OW in childhood and adolescence are conservative multi-component weight reductions programs with the aim of a long-term lifestyle change [10, 11]. The NICE [12] and German guidelines [13] for treatment and prevention of OB, as well as many reviews [14–16] state that these weight reduction programs should include sustainable interventions targeting nutrition, physical activity and behavior change, addressing the whole family. Treatment options like medication (favorably in combination with weight reduction programs) or bariatric surgery currently play a minor role in children and adolescents [17, 18]. Importantly, in state-of-the-art multi-component weight reduction programs, parents play an essential role as *agents of change* [19] since they are mainly responsible for shaping their child’s habits and life circumstances, hence parents or caregivers need to be (co)-addressed in therapy.

One important aspect of these multi-component treatments which is included in the German guidelines and in the guidelines of the American Academy of Paediatrics, is motivation [13, 20]. Treatment motivation is a well-known impact factor of success in medical treatment in general [21–23]. Motivation is especially relevant in long-term and chronic diseases such as OB and OW, as it majorly determines treatment uptake, behavior change and maintenance of change. Practitioners should set a focus on the improvement of treatment motivation in children/adolescents and their families.

There is a lot of literature and different theoretical frameworks about motivation in medical treatment whereof we want to explain the two most common ones in the following. Many programs aiming to change health behaviors are based on the Transtheoretical Model (TTM) by Prochaska & DiClemente [24] which posits that behavior change follows five Stages of Changes (SoC): *precontemplation, contemplation, preparation, action,* and *maintenance* [25]. The stages describe a person’s attitude, motivation and also behavior towards change, i.e., in *precontemplation* a person is not aware of advantages and disadvantages of the new and old behavior and has not (yet) developed the wish for change. *Maintenance*, as the last SoC, means having changed one’s behavior and now trying to prevent relapses. The TTM also involves *self-efficacy*, as belief in one’s own ability to change, the *processes of change* and the *pros and cons of change* [24, 26]. Beyond that, *Motivational Interviewing* (MI), which is derived from the TTM, is a specific method to enhance motivation [27]. MI is a conversation style to support clients who show ambivalence in terms of behavioural change towards a healthier behavior [27]. Clients are supported to find advantages in the change and to increase their intrinsic motivation. Systematic reviews found that MI results in better anthropometric and other health outcomes in weight reduction program in comparison to standard care [28, 29]. In addition, the general literature about MI recommends using *readiness rulers* as a therapeutic approach for decreasing ambivalence and increasing intrinsic motivation [30]. They function as a Visual Analogue Scale where patients should rate their current level of motivation on the three subscales readiness for change, importance of change and confidence with change [30].

Another influential motivation theory on which many programs base, is the Self-Determination theory (SDT) by Deci & Ryan [31]. It describes human behavior as depending on the satisfaction of the basic human psychological needs for competence, relatedness, and autonomy. Behaviors are intrinsically motivated if they satisfy these needs whereas extrinsic motivation describes a motivation which is influenced by external factors. While TTM and SDT seem to be the most widely applied motivation theories in weight reduction literature, other frameworks have also been used. For example, the Theory of Planned Behaviour states that our behavioural intentions are influenced by our attitudes, subjective norms, and perceived behavioural control [32].

To conclude, weight reductions programs for children and adolescents with OB and OW are urgently needed, and current guidelines recommend that these should also focus on treatment motivation. Previous reviews have examined the motivations for weight loss in adolescents [33], or have assessed the effectiveness of motivational interviewing (MI) [34]. At the same time, there is no comprehensive evidence on the role of motivation in weight reductions programs for children and adolescents. The aim of the present review was to systematically synthesize this evidence in order to clarify the role of motivation in weight reduction programs for children and adolescents. We divided the research questions into three sub-questions focusing firstly on the theoretical framework and secondly the use of motivation as outcome or predictor variable in the weight reductions programs:

- Theoretical framework and assessment of motivation

Which theoretical frameworks of motivation have been applied in weight reductions programs for children and adolescents with OB and OW? How is motivation assessed in the weight reduction programs?

- Motivation as outcome

How does motivation change during a weight reduction program?

- Motivation as predictor

How does (baseline) motivation influence the success of a weight reduction program in children and adolescents with OB and OW?

## Methods

The process of conducting this systematic review was according to the PRISMA guidelines [35]. The protocol for the review was pre-registered on the PROSPERO international prospective register of systematic reviews (submission ID: CRD42024517048).

### Literature search

Four databases (PubMed, PsycINFO, Web of Science and Cochrane) were used to perform systematic literature searches in September 2023. A general search term was developed and adapted to the demands of each database. MeSH terms were used if possible. The search term consisted of the following five components: parents, children/adolescents, obesity, weight loss, motivation. The search terms for the four databases can be seen in Table 1.

**Table 1:** Search terms used in the different databases

PubMed	(((("child*"[Title/Abstract] OR "kid"[Title/Abstract] OR "kids"[Title/Abstract] OR "teen*"[Title/Abstract] OR "youth*"[Title/Abstract] OR "adolescent*"[Title/Abstract] OR "son"[Title/Abstract] OR "sons"[Title/Abstract] OR "daughter*"[Title/Abstract] OR "boy"[Title/Abstract] OR "boys"[Title/Abstract] OR "girl*"[Title/Abstract] OR "infant*"[Title/Abstract] OR "pediatric*"[Title/Abstract] OR "offspring"[Title/Abstract] OR "infant"[MeSH Terms] OR "adolescent"[MeSH Terms] OR "child"[MeSH Terms]) AND ("overweight"[Title/Abstract] OR "obes*"[Title/Abstract] OR "adipos*"[Title/Abstract] OR "obesity"[MeSH Terms] OR "adiposity"[MeSH Terms] OR "Pediatric Obesity"[MeSH Terms] OR "Overweight"[MeSH Terms:noexp])) OR ("parent*"[Title/Abstract] OR "father*"[Title/Abstract] OR "mother*"[Title/Abstract] OR "caregiver*"[Title/Abstract] OR "legal guardian*"[Title/Abstract] OR "Legal Guardians"[MeSH Terms:noexp] OR "caregivers"[MeSH Terms] OR ("Parents"[MeSH Terms] NOT "Surrogate Mothers"[MeSH Terms]))) AND ("Weight Loss"[Title/Abstract] OR "weight management"[Title/Abstract] OR "weight intervention"[Title/Abstract] OR "healthy weight gain"[Title/Abstract] OR "weight reduction"[Title/Abstract] OR "weight control"[Title/Abstract] OR dieting[Title/Abstract] OR "lose weight" [Title/Abstract] OR "reducing weight" [Title/Abstract] OR "Weight Reduction Programs"[MeSH Terms] OR "diet, reducing"[MeSH Terms] OR "Weight Loss"[MeSH Terms:noexp])) AND ("motivation*"[Title/Abstract] OR "stages of change"[Title/Abstract] OR "readiness"[Title/Abstract] OR "willingness"[Title/Abstract] OR "transtheoretical model"[Title/Abstract] OR "transtheoretical model"[MeSH Terms] OR "Motivation"[MeSH Terms:noexp])
PsycINFO	**#1:** ( DE "Parents" OR DE "Fathers" OR DE "Mothers") OR TI ( parent* OR father* OR mother* OR caregiver* OR "legal guardian*") OR AB ( parent* OR father* OR mother* OR caregiver* OR "legal guardian*") **#2:** DE "Pediatrics" OR TI ( child* OR kid OR kids OR teen* OR youth* OR adolescent* OR son OR sons OR daughter* OR boy OR boys OR girl* OR infant* OR pediatric* OR offspring) OR AB ( child* OR kid OR kids OR teen* OR youth* OR adolescent* OR son OR sons OR daughter* OR boy OR boys OR girl* OR infant* OR pediatric* OR offspring) **#3:** ( DE "overweight" OR DE "obesity") OR TI ( overweight OR obes* OR adipos*) OR AB ( overweight OR obes* OR adipos*) **#4:** ( DE "weight loss" OR DE "weight control" OR DE “Diets”) OR TI ("weight loss" OR "weight management" OR "weight intervention*" OR "healthy weight gain" OR "weight reduction" OR "weight control" OR dieting OR “lose weight” OR “reduce weight”) OR AB ( "weight loss" OR "weight management" OR "weight intervention*" OR "healthy weight gain" OR "weight reduction" OR "weight control" OR dieting OR “lose weight” OR “reduce weight”) **#5:** ( DE "motivation" OR DE "stages of change" OR DE Transtheoretical model" OR DE "readiness to change") OR TI ( motivation* OR "stages of change" OR readiness OR willingness OR "transtheoretical model") OR AB ( motivation* OR "stages of change" OR readiness OR willingness OR "transtheoretical model") Strategy: (S1 OR (S2 AND S3)) AND S4 AND S5
Web of Science	**#1:** TS = (parent* OR father* OR mother* OR "caregiver*" OR "legal guardian*") **#2:** TS = (child* OR kid OR kids OR teen* OR youth* OR adolescent* OR son$ OR daughter* OR boy$ OR girl* OR infant* OR pediatric* OR offspring) **#3:** TS = (overweight OR obes* OR adipo*) **#4:** TS = (weight NEAR/2 (management OR intervention OR loss OR reduction OR control) OR "healthy weight gain" OR dieting OR “lose weight” OR “reduce weight”) **#5:** TS = (motivation* OR "stages of change" OR readiness OR willingness OR "transtheoretical model") Strategy: ((#2 AND #3) OR #1) AND #4 AND #5
Cochrane	**#1:** (parent* or father* or mother* or caregiver* or legal guardian*).ti,ab,kw. or Legal Guardians.sh. or Caregivers.sh. or exp Parents/ **#2:** (child* or kid or kids or teen* or youth* or adolescent* or son or sons or daughter* or boy or boys or girl* or infant* or pediatric* or offspring*).ti,ab,kw. or infant.sh. or adolescent.sh. or exp child/ #3: (overweight or obes* or adipos*).ti,ab,kw. or obesity.sh. or adiposity.sh. or pediatric obesity.sh. or overweight.sh **#4:** ((weight adj2 (loss or management or intervention or reduction or control)) or healthy weight gain or dieting or reduce weight or lose weight).ti,ab,kw. or exp weight reduction programs/ or diet, reducing.sh. or weight loss.sh **#5:** (motivation* or stages of change or readiness or willingness or transtheoretical model).ti,ab,kw. or transtheoretical model.sh. or exp motivation/ **Strategy:** ((2 and 3) or 1) and 4 and 5

### Eligibility criteria

Inclusion and exclusion criteria were defined according to the PICOS criteria [35] prior to the screening process and can be seen in Table 2.

**Table 2:** Pre-defined PICOS criteria and inclusion and exclusion criteria used in the screening process

	Inclusion criteria	Exclusion criteria
P participants	Parents of children or adolescents (2–21 years) with overweight or obesity (BMI > = 85th percentil)	• Older/younger children or adolescents • Parents with overweight or obesity without children (or no data on children) • Endocrinological or genetic comorbidities which induced the overweight (e.g., Prader–Willi syndrome) • Bulimia nervosa as comorbid eating disorder
I intervention	• Intervention study • Aim of the intervention: weight reduction or prevention of weight gain • Duration at least four weeks • Intervention should contain at least one of the three recommended therapy modules (nutrition, physical activity and behavior)	• Bariatric surgery as intervention • Medication as (only) intervention
C comparator	No control group needed	–
O outcome	• Any assessment of motivation as outcome or as predictor variable (no need for validated questionnaire) • Assessment at least once	Studies which do not assess motivation
S study design	• Longitudinal studies • Two or more measurement points (whereof motivation needs to be assessed at least once)	• Cross-sectional studies • Qualitative studies • Case studies

### Study selection

**Fig. 1:**
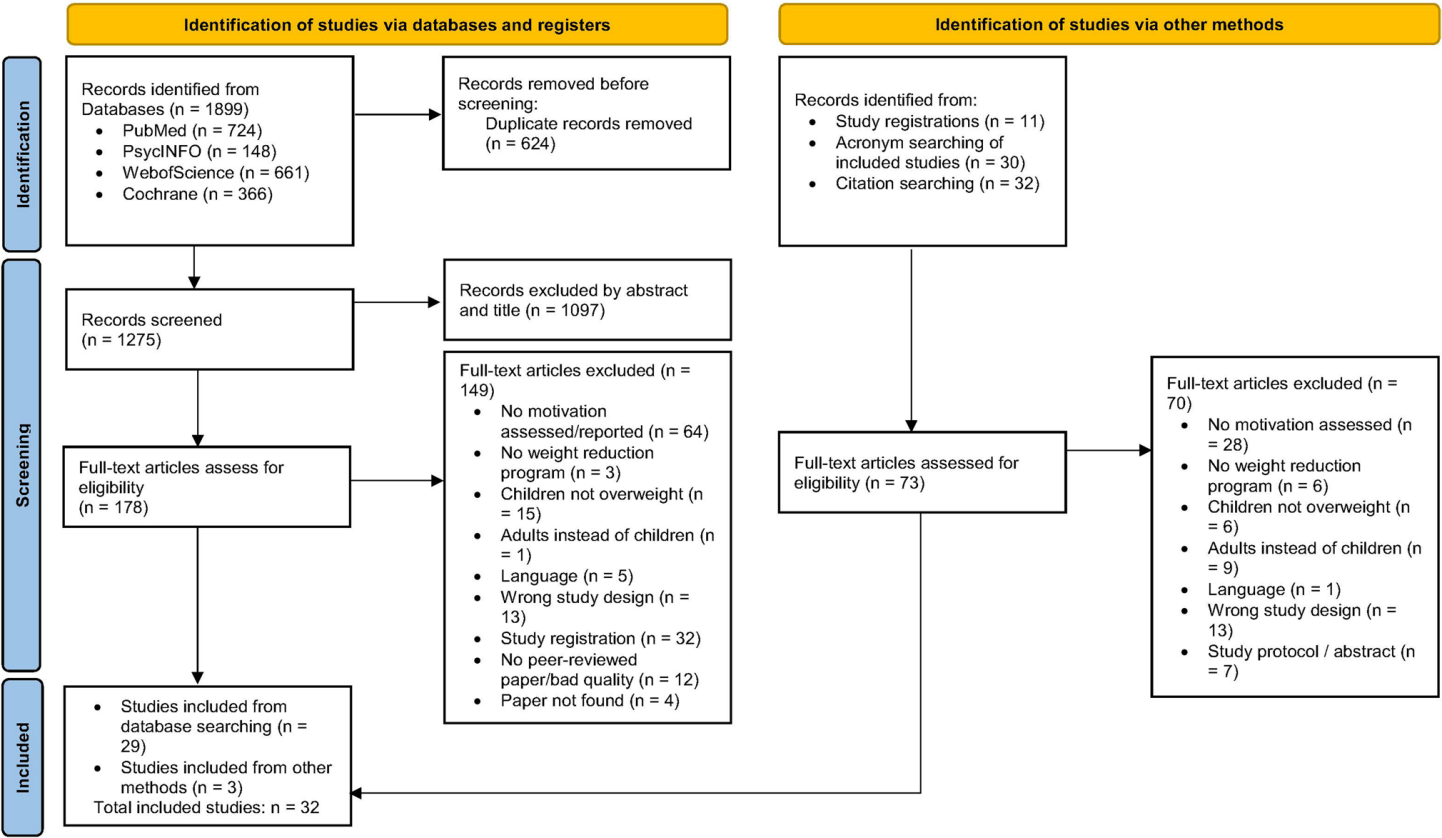
PRISMA flow diagram of the study selection process

The selection process is depicted in the flowchart (Fig. 1). The initial searches yielded 1899 results in total. After the removal of 624 duplicates, two independent researchers (AH and AW) screened titles and abstract of the remaining 1275 studies and rated the studies according to the eligibility criteria. Interrater reliability was good with *κ* = 0.62. In case of disagreement the articles were discussed and if no consensus was found, a third rater (KZ) was consulted. For the 178 articles proven eligible, the full text was read and eligibility was assessed by one rater (AH). 149 studies were excluded due to different reasons (see Fig. 1) and 29 articles were included. An additional hand search yielded 73 results which were assessed for eligibility. 70 reports were excluded and 3 were included resulting in a total of 32 reports included in this review [36–67].

### Data extraction

The 32 resulting studies were separated in two categories according to research questions two and three: Table 3 shows studies using motivation as an outcome variable to evaluate weight reduction programs. Table 4 shows studies that used motivation as a predictor, e.g., to find out if baseline motivation can predict weight loss or dropout. One study [42] is listed in both tables since it uses motivation as both, outcome and predictor. Two articles [36, 67] describe the same study, while one article describes pre-post data and the other article describes follow-up data. For all studies, authors name, year of publication, sample size, participant characteristics (age, gender, weight of children or adolescents), intervention description, and a description of the assessment of motivation were extracted from the studies. Further information was extracted for each research question separately and differ between Tables 3 and 4. Table 3 shows extracted variables with information on motivation as outcome variable, such as changes in motivation during the weight reduction program. Table 4 contains extracted variables with information about motivation being used as a predictor variable, such as the relationship between motivation and the outcome variable.

**Table 3:** Overview over studies using motivation as outcome variable

Authors, year	Sample size, mean age, sex	Baseline weight	Intervention description	Assessment of motivation	Results: change in motivation	Results: weight change
Boff et al., [36]	*N* = 135, • IG: 16.4 ± 1.2y, 44.4% girls; • CG: 16.5 ± 1.0 y, 55.6% girls	• IG: BMI: 34.74 ± 4.71 kg/m2, BMI percentile: 99.32 ± 0.87 • CG: BMI: 35.99 ± 6.51 kg/m2, BMI percentile: 97.88 ± 2.74	IG: 12 meetings (1.5 h) over 3 months, held by interdisciplinary team, 2 sessions with parents, based on TTM CG: 12 weekly meetings (1 h) with traditional health education, 2 sessions guided by multidisciplinary team, without TTM training, content: diet, physical activity (directive and non-motivational)	• readiness to change diet and to start exercise of adolescents (VAS) • self-efficacy to diet and to exercise of adolescents (VAS) • motivation to participate in MERC [study name] of adolescents (VAS) • decisional balance of adolescents (self-report instrument)	• readiness to change diet and to start exercises significantly increased over time within groups • self-efficacy for diet shows significant increase within groups (not in self-efficacy for exercise) • motivation to participate in MERC significant increase within groups over time • between groups only significant differences for decisional balance (increase in CG) over time	• significant mean reduction of BMI within groups over time • in IG significant decrease of BMI percentile over time, in CG decrease
Delamater et al., [37]	*N* = 24, 11.0 ± 1.2 y, 41.6% girls	BMI percentile: 98, zBMI: 2.19	4 weeks, web-based intervention (“families acting together for Health”) with games, texts, graphics, narration. Content: Stimulus control, modification of eating behavior, increasing physical activity, reducing sedentary behavior. Section with information for parents	Children’s intrinsic motivation (Intrinsic Motivation Inventory for Weight Management questionnaire with self-efficacy and importance subscales)	• Significant improvements in intrinsic motivation total score and self-efficacy subscale • Marginal interaction with usage: high users show higher increase in self-efficacy subscale	• no significant changes in zBMI pre-post • Interaction with program usage: high users reduced zBMI, low users increased zBMI over time
Fenner et al., [65]	*N* = 56	Not reported	8 weeks biweekly, multidisciplinary family intervention delivered by multi-professional team, beginning with physical activity for adolescents and educational information for parents, education for both in second hour; need-supportive environment; behavior-change techniques. Also parent-only sessions. Topics: expectations, recipe modification, overcoming barriers, parenting; goal-setting integrated in SDT	Autonomous motivation of adolescents: • For physical activity (revised version of Behavioral Regulation in Exercise Questionnaire Version 2 and Integrated Scale for Exercise Behavior) • For healthy eating (adapted version of Perceived Locus of Causality for Diet and Integrated Regulation Scale for Exercise Behavior)	• Significant improvements in autonomous motivation for physical activity and healthy eating after intervention • Significantly elevated point estimates in follow-up • Monthly rate of change in autonomous motivation during intervention in IG was significantly different to waitlist CG	Not reported
Fonvig et al., [38]	*N* = 129, 11.2y, 54.9% girls	BMI-SDS: 2.81	Outpatient, family-based, multidisciplinary program following “The Children Obesity Clinic Treatment protocol”. Start: 1-h consultation with pediatrician/pediatric nurse and creation of tailor-made treatment plan with behavior-modifying techniques, lifestyle and nutrition advices. Consultation again annually and every 6–8 weeks, if needed consultation with psychologist or social worker	Children’s motivation for weight loss (one question, VAS) as one domain of psychosocial well-being	After treatment (2–82 months) significant reduction in motivation between baseline and follow-up	• After 14 months median BMI-SDS reduction by 0.29 • Change in BMI-SDS positively associated with changes in motivation (only in older group)
Gourlan et al., [50]	*N* = 54, 13 ± 1.7y, 41% girls	• IG: BMI:29.56 ± 4.75 kg/m2 • CG: BMI: 29.59 ± 5.92 kg/m2	6 month intervention with standard weight-loss program (SWLP) and MI. IG: SWLP + MI, CG: SWLP SWLP: 2 individual 30 min-face-to-face sessions with a specialized doctor over 3 months. Focus: Physical activity; consequences of physical activity in general and to the individual, goal setting, when and where to perform the behavior, self-monitoring, feedback on the behavior MI: 6 20 min phone sessions with trained physical activity counsellor over 6 months. Sessions followed a semi-structured format including 4 phases: (1) acquaintance and building awareness, (2) alternatives and problem-solving, (3) goal and agenda setting, (4) behavior modification consequences and perspectives	• Adolescents’ motivation for physical activity (French version of Behavioral Regulation Exercise Questionnaire, BREQ-2) • Adolescents’ perceived Competence (confidence in ability to engage in physical activity) • Adolescents’ perceived autonomy support (French version Health Care Climate Questionnaire)	• Significant increase in intrinsic motivation in CG (not in IG) • IG: significant increase in integrated and identified regulation • IG: significant decrease in amotivation	• No change over time in BMI in CG • Significant decrease of BMI in IG • No difference between groups in follow-up
Gourlan et al., [49]	*N* = 18, 14.3 ± 1.7 y, 77,8% girls	BMI: 33.47 ± 3.11 kg/m^2^	11 week local residential treatment program: supervised weekly exercise sessions (based on concept of autonomy support [SDT], e.g., received feedback after each session and expressed their feelings about difficulties.), moderate dietary restriction and nutrition education	Adolescents’ motivation for physical activity (French version of the exercise motivation scale, assessing five motivational constructs), Relative Autonomy Index was used as outcome	• Significant increase in Relative Autonomy Index (higher scores indicating more autonomous motivation) between baseline and end • Tendential increase (*p* < .10) in intrinsic motivation and tendential decrease in external motivation between baseline and end	Significant BMI reduction between baseline and end of program
Ham et al., [48]	*N* = 75, • IG: 10.8 ± 1.2, 43.7% girls • CG: 10.3 ± 0.9 y, 44.4% girls	• IG: BMI: 24.35 ± 2.73 kg/m2 • CG: BMI: 24.22 ± 2.24 kg/m2	IG: TTM-based exercise counseling by trained nurse, eight 30 mins individualized counseling sessions over 3 months, in school, TTM-counseling booklets, program adapted to needs, based on SoC. Topics of counseling: Information on obesity, self-image, problem health behaviors, overcoming barriers and associated efforts, commitment to change, parental involvement, self-reflection CG: counseling booklets, one session counseling Both groups: weekly, 60 min music skipping rope exercise led by professional instructor with phone calls/texts one day before	• SoC in exercise of children: one question for classification to one stage • Pros and cons of exercise (questionnaire for children) • Self-efficacy for exercise (questionnaire for children)	• 36.2% of IG and 17.4% of CG advanced their SoC of exercise behavior by at least one stage, but group difference was not significant • No significant change of pros and cons of exercise in both groups • Significantly increased self-efficacy in the IG, but not in CG	CG: significant increase in BMI at posttest; IG: maintenance of BMI
Jørgensen et al., [39]	*N* = 120, 10.4 ± 2.8 y, 53.3% girls	BMI-SDS: 3.1 ± 0.7	Family-centered multifactorial lifestyle intervention following “The Children's Obesity Clinic's Treatment protocol”. 6–8 visits per year at community healthcare offices, followed for maximum 3 years in total. Behavioral change techniques; individualized treatment plan at baseline	Children’s motivation for weight loss (one question, VAS) as one domain of psychosocial well-being	Significant decrease in motivation	• 68.5% of participants reduced BMI-SDS • Participants who achieved weight loss, experienced reduced motivation; participants with no change in BMI-SDS experienced loss of motivation
Kahana et al., [46]	*N* = 79, 51% girls, • IG: median age: 9 y • CG: median age:10 y	• IG: BMI median: 25.4 kg/m2, BMI percentile median: 99 • CG: BMI median:26.0 kg/m^2^, BMI percentile median: 99	5-month weight management program (CG) with additional exergame App (IG). Both groups received weekly text messages to remind them to be active for at least one hour per day. Program consisted of 2 1 h physical activity sessions per week, 3–5 group sessions with dietitian, 12–23 group sessions for parents with dietitian and social worker, healthy cooking workshop, combined children-parents physical activity session, 2–3 individual sessions with a dietitian (children only) and 2 sessions with a physiotherapist to set goals for physical activity. Additional for IG: access to 2 physical activity apps and one of the weekly activity sessions included activity with an app	Attitude towards physical activity, relating to types of motivation (Behavioral Regulation in Exercise Questionnaire, BREQ-2 for children)	CG: significant improvement of Relative Autonomy Score of BREQ (higher score means more autonomous motivation); IG: no significant improvement	• No difference between groups pre and post-intervention • BMI decreased in both groups
Kotler et al., [40]	*N* = 16, 13.2 ± 1.1 y, 62.5% girls	BMI: 36.8 ± 5.5 kg/m^2^	6 week multidisciplinary summer day-treatment program. Content: academics, physical exercise, recreation facilities, nutrition education, cooking groups, behavioral therapy, art therapy, yoga. Focus on healthy eating and physical activity, not explicit on weight loss. Weekly educating parent group	Motivation for weight change of adolescents, assessed with 2 questionnaires: • stages of change algorithm • University of Rhode Island Change Assessment Scale	• Stage of change algorithm: baseline 81% in contemplation, 19% in action; at week 6 19% in contemplation, 75% in action, 6% in maintenance • University of Rhode Island Change Assessment Scale: increase in scores on action	No significant changes in BMI over time (10 subjects decreased BMI, 6 subjects increased BMI)
Lee et al., [41]	*N* = 104, 11.0 ± 2.1y, 35.6% girls	• IG: zBMI: 2.27 ± 0.48 • CG: zBMI: 2.27 ± 0.51	24 weeks with 6 visits for both IG and CG, Nutritional intervention using “nutrition care process”, Topics of nutrition education: food groups, eating healthy proteins, five colored vegetables and fruits, food portion control, nutrition facts label, high-calorie low nutrition food; education was performed by nutritional expert. Only for IG: at every visit additional individualized NCP by nutritional expert in one-to-one setting	• Stage of change in children (assessment not described) • Self-efficacy in children (with sub-categories motivation, understanding and prediction of change) questionnaire	• Self-efficacy significantly increased in IG • End of intervention: 30.2% of IG were in maintenance stage, 16% of CG	• Self-efficacy negatively associated with zBMI • zBMI significantly decreased in IG
Luca et al., [42]	*N* = 75, • IG: 15.1 ± 1.8 y, 65% girls • CG: 14.9 ± 2.0 y, 59% girls	• IG: 44.8 ± 7.8 kg/m2, zBMI: 4.5 ± 1.1 • CG: 34.8 ± 8.0 kg/m2, zBMI: 3.1 ± 1.1	2 year-interdisciplinary program with 6 weeks weekly sessions, then biweekly for 6 months and follow-up. Topics provided by different experts promoting small sustainable health behavior changes, individual and group sessions (for parents and adolescents). Content: nutrition, physical activity, stress management, strong focus on mental health	• Readiness to healthy lifestyle change (Readiness to change questionnaire, assessed from teen and parent) • Readiness to change for healthy dietary behaviors (questionnaire completed by dietitian in IG; in CG self-completed)	• IG: teen and parent scores in readiness increased at 6 and 12 months (in CG increase at 6 months) • Increase in dietary readiness at 6 and 12 months in IG	• After 6 months no change in BMI in IG; increase of BMI in CG (no significant difference in change between groups) • After 12 months increase in both groups (IG: trend, CG: significant)
MacDonell et al., [66]	*N* = 44, 15.1 ± 1.4 y, 79.5% girls	BMI: 35.36 ± 7.8 kg/m^2^, BMI percentile:96.74 ± 4.26	IC and CG matched for dose, timing and interventionist: 60-min session with youth and caregiver by a dietitian at weeks 1, 2, 6, 10 IG: using MI techniques, devising a change plan, discussing barriers, etc CG: nutrition counseling following a manual	Intrinsic motivation for nutrition and activity (self-regulation questionnaires; not described by whom answered)	Significant increase in intrinsic motivation for physical activity in intervention group (but decrease in actual physical activity)	No significant change in BMI in both groups
Schiel et al., [45]	*N* = 140, 13.7 ± 2.5 y, 55.7% girls	BMI: 30.5 ± 5.6 kg/m^2^, BMI-SDS: 2.44 ± 0.55	2 Phases: (1) 4–6 weeks hospital, (2) interactive telemedicine support program. Phase (1): Structured treatment and teaching program with 49 sessions by experts and outdoor activities. Parents received a 3 days program. Phase (2): After 4 weeks, 3,6,9 and 12 months participants received reminders and completed questionnaires. If the results suggested the need for action, an additional intervention (via internet or phone) was offered	Motivation of adolescents (questionnaire not described)	In phase 2 decrease in motivation	• Significant BMI-SDS reduction after phase 1 • Stabilization of BMI-SDS after phase 2
Segalla et al., [67]	*N* = 135, 16.4 ± 1.1 y, 74.1% girls	• IG: BMI: 34.7 ± 0.58 kg/m2 • CG: BMI: 36.0 ± 0.77 kg/m2	12 week interdisciplinary intervention program with psychoeducational CG and motivational IG. For both groups: 12 weekly sessions over a 3 month period by experts. Additionally, one education session for the caregivers CG: conduct educational actions and engage in discussion with the adolescents regarding factors of health promotion, quality of life and risk IG: additional to topics of CG: motivational approach and TTM theoretical basis to develop empowerment and autonomy for change	• Adolescents’ readiness-to-change in lifestyle (readiness-to-change-scale: two questions for physical activity and nine questions for eating behaviors), VAS • eating habits self-efficacy scale (questionnaire for adolescents) • decisional balance for weight loss (questionnaire for adolescents)	• significant increase in readiness in both groups over time • interaction effect between group and time for decisional balance: increase in IG at t2 and for CG at t1 • mixed results for various subscales of self-efficacy	no significant change over time in BMI in IG, but increase to follow-up in CG
Van Rinsum et al., [44]	*N* = 41, 10.2 ± 3.5 y, 56.1% girls	zBMI: 2.4 ± 0.4	8–10 months combined lifestyle and health intervention with coaching by trained lifestyle coaches, 8 group sessions and 4–10 individual sessions. Topics: physical activity, dietary behaviors, sleep. different programs, depending on the age	Motivation for physical activity (Behavioral Regulation of Physical Activity in Children Scale), assessed by children from the age of 10	no significant changes from baseline in motivational subscales	No significant change in zBMI after intervention
Verloigne et al., [47]	*N* = 65 15.5 ± 1.4 y, 59% girls (subsample for study aim suitable to review question)	BMI: 35.9 ± 5.7 kg/m^2^, zBMI: 2.62 ± 0.4	10 month multi-component program with residential weight reduction treatment in local centers with physical therapists and educators. Moderate dietary restriction, regular physical activity in peer group (4 h per week, 2 h physical education, 2 h supervised games per day), cognitive behavioral techniques. Received also group and individual psychological support and medical supervision without medication. Fulfillment of the needs autonomy, competence and relatedness [SDT]	Motivation towards exercise in adolescents (behavioral regulation in exercise questionnaire, BREQ-2)	• Significant increase in composite score of relative autonomy over time • Significant increase in autonomous motivation, external, introjected, identified and intrinsic regulation over time	Not reported
Watson et al., [43]	*N* = 63, 14.8 ± 2.3 y, 100% girls	BMI: 29.2 ± 5.1 kg/m^2^	12 week interventions with 3 groups: 1) recommended diary diet (4 servings of diary a day) plus exercise (RDa), 2) low-diary diet (maintain low diary consumption) plus exercise (LDa), 3) control. RDa and LDa: 1 h dietary counselling 5 times by expert, exercise 60–90 min training three times per week either individually or in groups of 2–3 (depending on preference), participants were provided with a Fitness Watch to use especially for non-training days. RDa and LDa intervention delivery underpinned by SDT	• motivation for healthy eating and motivation for exercise of adolescents (two versions of treatment self-regulation questionnaire) • confidence to maintain a healthy diet and to exercise regularly of adolescents (perceived competence scale)	Diet-related: • Positive motivational profile in all groups at both time-points • No between-group differences in 0–12 week changes in motivation and perceived competence • Decrease in amotivation for one IG, increases in perceived competence in other IG Exercise-related: • Positive motivational profiles in all groups at baseline and post-intervention • No between-group differences in 0–12 week changes in motivation and perceived competence • CG increase in autonomous motivation • One IG shows increase in perceived competence	Not reported

y: years; VAS: Visual Analogue Scale; IG: intervention group; CG: control group; MI: Motivational Interviewing

**Table 4:** Overview over studies using motivation as predictor variable

Authors, year	Sample size, age, sex	Baseline weight	Intervention description	Assessment of motivation	Main outcome measure /measurement of success	Results: relation between motivation and outcome
Accurso et al., [64]	*N* = 50, 10.9 ± 1.3 y, 62% girls	BMI: 24.6 ± 2.7 kg/m^2^, BMI percentile: 95.1 ± 2.9	5 month guided self-help weight-loss program with 11 20 min sessions and 1 60 min session by clinical psychology doctoral trainees. Content: monitoring child and parent weight, self-monitoring booklets on food intake and physical activity and barriers to implementing program recommendations. Sessions were with children and parents, but focus was on parents	Parent and child motivation before each session (each assessed by parent and interventionist one-item-rating on VAS)	BMI reduction	• no difference in early motivation between dropouts and completers • interventionist-rated child motivation only significant motivational predictor for child BMI change • rate of BMI decline steeper for children with higher interventionist-rated motivation
Braet et al., [61]	*N* = 72, 10.5 ± 2.6 y, 62.5% girls	Adjusted BMI: 163.89%	12 week non-diet healthy lifestyle program with 6 biweekly individual/group sessions. Followed by monthly follow-up meetings for 4–8 months after the last session, focus in follow-up not only on children but also on parents to motivate the family to continue. Focus: healthy eating habits, moderate exercise, cognitive behavioral techniques. Topics: food and exercise diaries, goal setting, self-regulation, problem-solving skills	Motivation for treatment of parents and children (estimated by intake team, categorized as low, moderate, strong)	completion of the healthy lifestyle program (completers and non-completers)	• 47% of children ended prematurely • Significantly more strong motivation in parents of children who completed the treatment • No difference in motivation of children
Da Silva et al., [52]	*N* = 164, 13 ± 2 y, 53.1% girls	BMI: 30.71 ± 4.68 kg/m^2^	16 week multidisciplinary intervention with three 2 h sessions a week. First hour: group educational activities in physical education, nutrition or psychology held by professionals. Afterwards active engagement in physical activity while wearing a heart-rate-monitor. Additional individual meeting with pediatrician and family. Parents had appointments with the professionals at baseline and once a month	Readiness for behavior change for food and physical activity (stages of change questionnaire for adolescents)	• Outcomes of the weight reduction program: anthropometric parameters, body composition, physical fitness • adherence	• No association between SoC for physical activity and general domain and adherence • participants on maintenance stage reached more positive outcomes in most anthropometric measures, diastolic blood pressure and cardiorespiratory fitness
Dhuper et al., [53]	*N* = 596, no age reported, 58% girls	Not reported	Local pediatric lifestyle-modification program with enrollment in exercise classes at local community centers, held by certified trainers. Those classes were available Monday until Saturday at 6 centers, families were required to attend at least 2 classes per week. No time limit, families could participate, as long as they wanted (long-term group: 24 months or longer)	Maternal readiness (Parental Readiness Questionnaire)	Compliance: three categories based on attendance to fitness classes (low/medium/high attendance)	• generally high levels on self-reported readiness but low compliance • no significant differences between compliance categories regarding to readiness
Ehrmann et al., [54]	*N* = 146, • Completers: 14.4 ± 1.7 y, 69% girls • Non-completers: 14.2 ± 1.8 y, 66% female	• Completers: 43.9 ± 11.3 kg/m2 • Non-completers: 43.0 ± 10.5 kg/m2	6 months multidisciplinary weight management program with family-focused weekly aerobic and strengthening activities, biweekly nutrition group classes, monthly individual group behavioral session (motivation and goal setting); based on SDT, MI and behavior change strategies. Delivered by experts	Adolescents’ readiness and Confidence (standard readiness rulers for each, with VAS)	Completion of the weight management program: completers and non-completers (dropout any time after joining)	• 53% of adolescents completed the program • No relationship between either readiness or confidence and program completion
Gunnarsdottir et al., [55]	*N* = 84, 11.0 ± 1.4 y, 45.2% girls	• Completers: BMI-SDS: 3.12 ± 0.5 • Dropout: BMI-SDS: 3.10 ± 0.5	18 weeks of Epstein's Family-based Behavioral Treatment for children + one parent with 12 individual family sessions and 12 group sessions. Group sessions: 60–90 min, separately for children and parents. Individual sessions: 20 min with weighing and reviewing of habit books/self-monitoring. Additionally provided with reading material and physical activity program; content: information about weight control, self-monitoring, goal-setting, behavior change techniques, and maintenance of behavior change	Parental motivation conceptualized as importance, confidence and readiness (questionnaire for parents)	• Treatment completion • weight change at early response (five weeks) • weight change at late response (treatment end) • weight change at follow-up (1 year)	• confidence significantly lower in parents who dropped out than in completers • confidence predicted weight change at early response and at treatment end (but not at follow-up) • importance and readiness predicted neither completion nor weight loss at any stage (but scores generally high)
Luca et al., [42]	*N* = 75, • IG: 15.1 ± 1.8y, 65% girls • CG: 14.9 ± 2.0y, 59% girls	• IG: 44.8 ± 7.8 kg/m2, zBMI: 4.5 ± 1.1 • CG: 34.8 ± 8.0 kg/m2, zBMI: 3.1 ± 1.1	See Table 2	• Readiness to healthy lifestyle change (Readiness to change questionnaire, answered by teen and parent) • Readiness to change for healthy dietary behaviors (questionnaire completed by dietitian in IG; in CG self-completed)	Reduction of BMI at 12 months	lower parental readiness to change score at baseline, as well as higher teen dietary readiness score associated with greater BMI reduction
MacDonell et al., [51]	*N* = 24, 14.5 ± 1.7y, 75% girls	BMI: 38.5 ± 9.3 kg/m^2^	6 months family-centered, community based treatment (multisystemic Therapy). Approximately 2–3 therapy sessions per week (for 6 months) by therapists. 4 Key features of the treatment: (1) risk factors, (2) empirically based clinical treatments, (3) focus on promoting behavioral changes in the youth's natural ecology, (4) home-based model. Incorporated strategies: self-monitoring of food intake and physical activity, environmental control, setting specific weight-loss goals, caregiver reinforcement of successful behavior change	Motivation to change (readiness) by adolescent and caregiver (Rollnick’s readiness rulers for different behaviors critical to weight loss, VAS)	Weight change measured in zBMI (in association with treatment dose)	• Number of sessions significantly correlated with weight change and BMI change • Adolescents’ readiness significantly associated with treatment dose
Mâsse et al., [58]	*N* = 160, 13.2 ± 5.9 y, 57.2% girls	BMI: 30.7 ± 5.9 kg/m2, zBMI: 2.67 ± 0.82	8 months web-based lifestyle intervention with adolescents and one parent. They accessed the program from home and returned for in-person assessments at 4 and 8 months. Intervention: focused on teaching healthy habits (increasing fruit and vegetable intake, decreasing dietary fat intake, increasing total physical activity, and reducing screen time). Adolescents received individualized Web-based weight management with corresponding tailored intervention for their parents to provide them with strategies to support their adolescents. The intervention was initially developed as a 1-year intervention with three 16-week phases, but this study implemented only the first two phases. Motivational phone interviews. Intervention included elements from social cognitive theory and TTM	• theory of planned behavior constructs: questionnaire for adolescents (attitude, subjective norms, perceived behavioral control, intention) • SDT constructs: questionnaire for adolescents (intrinsic motivation, autonomy support, relatedness)	Adolescents’ adherence operationalized as: • use of website • completion of counselling calls • use of tracking tools	• intrinsic motivation key predictor to use of website • intrinsic motivation predicted adherence • autonomy control and relatedness predicted adherence indirectly through intrinsic motivation • Constructs of SDT predicted adherence, but not constructs of theory of planned behavior
Naar et al., [59]	*N* = 181, 14.3 ± 1.4 y, 67% girls	BMI: 38.15 ± 7.45 kg/m^2^	Phase 1 (3 months): randomly assigned to either home- or office-based cognitive-behavioral and MI family-based weight-loss treatment. 2 weekly sessions by community health workers. Topics for this phase: goal development, nutrition, physical activity, building behavioral weight loss skills (both for adolescents and caregivers) Phase 2 (3 months): Nonresponders of phase 1: randomly assigned to either continued skills training or contingency management, responders received relapse prevention	Adolescents’ initial motivation with dimensions of self-efficacy/confidence for making lifestyle changes and perceived importance of making lifestyle changes (Rollnick’s Readiness Rulers for 15 items, VAS)	Percent weight loss (percentage over median age- and gender-normed BMI)	• higher initial confidence associated with more reduced percentage overweight • confidence moderated phase 2 treatment • perceived importance no significant moderator
Reinehr et al., [60]	*N* = 75 • successful: 11.6y, 46% girls • no success: 11.8y, 52% girls	• successful: BMI: 28.8 kg/m2, BMI-SDS:2.5 • no success: BMI: 30.4 kg/m2, BMI-SDS: 2.7	One year outpatient training Obeldicks with 4 phases, each phase 3 months: 1 Exercise therapy (1–2 times a week), nutritional course (2 times a month), behavior therapy (2 times a month, includes, e.g., contingency contracting), Parents group session (2 times month). 2 Exercise therapy (1–2 times a week), individual psychological therapy, talk round for parents (1 time a month). 3 Exercise therapy (1–2 times a week), individual psychological therapy. 4 Exercise therapy (1–2 times a week). Delivered by interdisciplinary team	Willingness to change behavior operationalized as: • number of previous therapy trials • number of previous participations in exercise groups • changes in weight status in last three months	Success: Reduction of BMI-SDS at end of training	Previous participation in exercise groups only significant factor of willingness associated with success
Rodriguez et al., [56]	Screening of *N* = 19634; invitation sent to *N* = 2862 • decline: *N* = 837, 13.2 ± 0.4 y • accept: *N* = 2025, 13.2 ± 0.5 y	• decline: 26.7 ± 2.89 kg/m2 • accept: 26.7 ± 2.8 kg/m2	Sent out invitations to families with children with BMI > 90 (data from former study) and invitation to participate in MBIOM (Multi-component-intensive-behavioral-intervention for obesity management) study with training sessions. Engagement in this study was measured (see outcome)	Initial readiness for behavior change (SoC) and willingness to make lifestyle changes; both by interview of parents	• Uptake of weight reduction program (acceptance of invitation) • engagement (participation in min two sessions)	• 29.24% declined invitation • 86.08% engagement in two or more sessions • Initial readiness to change was unimportant for engagement • Other differentiating factors were mostly found at uptake rather than engagement
Rojo et al., [63]	*N* = 165, 10.3 ± 1.4 y, 42.2% girls	zBMI: 3.13 ± 1.37	12 week program with 3 groups. Focus of the program: awareness, motivation to change, behavioral techniques, emotions, thoughts, body image, social skills, management of peer teasing, self-esteem and relapse prevention. Delivered experts CG: standard behavioral treatment with 4 individual monitoring sessions, aimed at health behavioral changes, delivered through MI ENTREN: Cognitive-behavioral treatment with content of CG + psychological workshop for children (10 sessions) + 2 nutrition and physical activity educational sessions ENTREN-F: Family-based cognitive-behavioral treatment with content of ENTREN + family workshop (6 sessions). Aim of family workshop: improving family awareness about the comorbidity risks of obesity and family functioning	Level of importance and self-perceived readiness of mothers (two questions with VAS)	short-term adherence	level of importance and readiness not associated with attendance
Taylor et al., [57]	*N* = 271 • participants: 6.4 ± 1.5 y, 56% girls • non-participants: 6.4 ± 1.4 y, 53% girls	• participants: zBMI: 1.63 ± 0.47 • non-participants: zBMI:1.56 ± 0.41	RCT involving feedback of weight status after screening followed by a 2-year behavioral intervention. Invitations were sent to families in several primacy care practices/clinics. Then families with children affected by OB/OW received feedback about children’s weight status with MI or usual care. After that families with children affected by OB/OW were invited to complete an interview 1–2 weeks after the health check. At the end of the interview, parents were invited to participate in a 2 year family-based lifestyle intervention	Parents’ motivation for healthy lifestyle in their children (modified treatment self-regulation questionnaire and motivational screening measure)	Recruitment in weight management program (uptake or no uptake)	• 72.7% of the families with children affected by OB/OW participated in intervention • Participating parents scored higher in introjected motivation; no differences in autonomous motivation between participating and non-participating families • Participating families were more likely to have tried to improve child’s diet, exercise and weight in past than non-participating families
Van Allen et al., [62]	*N* = 42, 4.6 ± 1.0 y, 54.5% girls	zBMI: 2.41 ± 0.61	IG: 6-month family-based preschool weight control intervention with 2 phases. (1) Intensive Intervention: 12 weekly sessions, alternating between group-based clinic sessions and individual home visits. (2) Maintenance: same concept as phase 1, but biweekly over 12 weeks. Topics of parents group-sessions: dietary education, physical activity and parenting skills. Manualized parent group by psychologist. Content of children group-sessions: nutrition education, structured meal, physical activity. Children group by pediatric psychologist. Home sessions were designed to support generalization of the clinic-taught skills to the home environment CG: enhanced standard of care; one individual pediatrician visit following a manual for each family, where BMI was explained and individually assessed and recommendations for exercise, diet and media use were made. Families received brochure ≥ Both groups taken together for this analysis	Changes in parental motivation during intervention (adapted version of Parent Motivation Inventory)	Changes from baseline to follow-up in: • zBMI • dietary variables • physical activity	• increase in parental motivation during intervention predicted reduction in zBMI of child, decrease in sweet and sugar sweetened beverages consumption 6 months posttreatment • increase in parental motivation during intervention predicted increase in artificially sweetened beverages 6 months posttreatment • change in parental motivation from baseline to posttreatment significantly associated with change in zBMI of child from posttreatment to follow-up

### Risk of bias in included studies

To assess the risk of bias in individual studies, we conducted a quality rating using the Quality Assessment Tool for Quantitative Studies by the Effective Public Health Practice Project (EPHPP) [68, 69] which is recommended for all quantitative studies. The tool consists of ratings on the categories study design, selection bias, confounders, blinding, data collection, and dropouts. Each category is built of two or three items. The items and categories are coded as *strong*, *moderate* and *weak*. A total rating for each article is based on the category ratings. Studies are rated overall *strong* (i.e., having a low risk of bias) if they have no categories with weak ratings, overall *moderate* with at most one *weak* rating and overall *weak* (i.e., having a high risk of bias) if two or more categories are rated as *weak*.

## Results

### Study and participant characteristics

The 32 included studies were published between 2003 and 2023. Most studies (*n* = 12) were conducted in Europe, nine studies in the US, three studies in Brazil and Canada, respectively, two in South Korea and one each in Israel, Australia and New Zealand. All studies were published in English. Sample sizes ranged from 16 to 2025 participating children and adolescents (*M* = 170.74, *SD* = 360.47, *Md* = 75). Their mean age was 12.13 years (*range* = 4.6–16.5 years) and there were more girls in the overall sample (57.12%). A detailed description of the 32 studies can be found in Tables 3 and 4.

### Theoretical framework and assessment of motivation

The included studies used different theoretical frameworks of motivation. Most studies referred to the TTM or the SDT (see introduction). Some studies [36, 48, 67] were strongly oriented towards the TTM, explaining its rationale, using TTM-specific methods [36, 48] such as consciousness raising or self-reevaluation in the intervention and using specific questionnaires to assess different components of the TTM. Boff, et al. [36] for example used the concepts of readiness to change, self-efficacy and decisional balance as outcome measures and asked participants to rate their current motivational state on Visual Analogue Scales. Ham, et al. [48] additionally used the SoC as well as pros and cons of change each specifically related to exercise as their outcome. The initial SoC was assessed by interview in Rodriguez, et al. [56] and by two specific questionnaires in Kotler, et al. [40]. Other studies (e.g., [40, 42]) used TTM-specific questionnaires without further explanation or use of TTM-specific methods in their intervention. Using MI as a motivation-enhancing method, some of the included studies compared the use of MI in their intervention with an intervention without MI [50, 66]. For example, adolescents and caregivers of the intervention group in the study of MacDonell, et al. [66] received four sessions in which behavior change in nutrition and physical activity was addressed. In these sessions MI techniques were used to elicit change talk. Intrinsic motivation for physical activity significantly increased in the intervention group. Regarding the construct of readiness rulers, some included studies used the same subdivision of motivation as applied in the readiness rulers (readiness, importance, confidence). In some studies (e. g. [51, 55]) motivation was subdivided in these aspects which then were used as outcomes or predictors of weight-loss treatment.

The second common motivation theory which is used in many included studies is the SDT: some authors explain its theoretical background, used parts in the intervention and used suitable questionnaires to assess the outcome [47, 49, 50, 65]. The most commonly used questionnaire was the *Behavioural Regulation in Exercise Questionnaire-2* (BREQ-2) [70] which measures motivation towards exercise. Other authors [44, 46] used suitable questionnaires without explaining their rationale or using components of it in their intervention.

Additionally, two studies described motivation as one component of psychosocial well-being [38, 39]. Lastly, there are also studies not using a certain construct or definition of motivation (e.g., [62]) and assessing the outcomes with questionnaires only using the term *motivation*. One study [60] operationalized motivation (defined as willingness to change in the reported study), with a more robust criterion, which was the number or previous therapy trials. To sum up, the authors base their weight reduction programs on very different theoretical frameworks and use different assessment methods and questionnaires.

### Motivation as outcome

18 of the included studies investigated motivation as an outcome variable (Table 3). Weight reduction programs for children and/or adolescents were evaluated and motivation was used as outcome variable, mostly next to weight change. The duration of the weight reduction programs varied (4 weeks until 2 years) while the content was mostly comparable, consisting of a multidisciplinary approach of nutrition, physical activity and behavior/education. Only the intervention in one study focused exclusively on nutrition [41]. Most studies only assessed the motivation of the participating child or adolescent. Only one study [42] additionally assessed parental motivation and used an expert-rating.

Most of the studies (*n* = 13) found an increase in motivation after as compared to before treatment. Kotler, et al. [40], for example, assessed motivation of adolescents with questionnaires based on the TTM. They found that at baseline there were 81% of participants in the (lower) SoC *contemplation* and 19% in the (higher) SoC *action* and after treatment after 6 weeks there were 19% in *contemplation*, 75% in *action* and 6% in *maintenance*. Gourlan, et al. [50] who based their work on SDT, found an increase in intrinsic motivation and a decrease of amotivation at the end of the program. Increases in motivation were found in studies which used MI or other motivational methods (e.g., [66]), as well as in studies using or reporting no motivation-enhancing methods (e. g. [40, 41]). Some studies also found an increase of motivation in the control group which received a different (reduced) intervention [36, 46, 50, 67]. Contrary to that, three studies [38, 39, 45] found a decrease in motivation. No change in motivation between pre and post-intervention was found in two studies [44, 46] but as a summary of these data, we can find an increase in motivation over the course of the programs in the majority of the included studies (*n* = 13). More information can be gained from Table 3.

Next to motivation researchers commonly analyzed weight change as common outcome in these weight reduction programs. Only three studies [43, 47, 65] did not report weight data in the present papers. Beyond the other studies, in seven [36, 38, 39, 41, 45, 46, 50] a reduction of weight/BMI/BMI-SDS/BMI percentile was found, six studies [37, 40, 44, 48, 51, 67] reported no change and one study found an increase [42]. The study of was the only one to report an association between weight and motivation: in the group of older children, a positive association between changes in BMI-SDS and changes in motivation was found. Since all other studies did not link weight change to motivational change we searched through the studies and tried to find associations. Beyond the studies reporting an increase in motivation, there were four studies finding a decrease in weight parameters [36, 41, 49, 50], five studies with no change [37, 40, 48, 51, 67] and one study with a weight increase [42]. Beyond the three studies reporting a decrease in motivation, all of them also reported a decrease in weight parameters [38, 39, 45]. Summarizing this, it is not possible to draw a clear conclusion about a possible association between weight change and motivation change over the course of a weight reduction program.

### Motivation as predictor

15 studies investigated motivation as a predictor (see Table 4). The weight reduction programs of the included studies lasted 12 weeks until 2 years and were all multidisciplinary. Motivation was investigated as a predictor of different outcomes. Outcome variables were heterogeneous between the included studies. They can be grouped into weight loss at different time points, adherence, dropout or the uptake of a weight reduction program. Most studies used more than one outcome variable and more predictor variables, such as different subscales of motivation. In contrast to the studies described before, here most studies assessed parental motivation [53, 55–57, 61–63] or parent and child motivation [42, 51, 64] and not only child/ adolescent motivation.

Eight studies reported mixed results regarding motivation as a predictor, i.e., some subscales of motivation or only the assessment of specific persons (e.g., parents or children/adolescents) predicted outcomes. Luca, et al. [42] for example assessed the readiness to a healthy lifestyle change with a questionnaire filled out by adolescents and parents, as well as the readiness for change for healthy dietary behaviors which was assessed with a questionnaire completed by a dietitian. They found that lower general parental readiness at baseline and higher dietary readiness of adolescents were associated with greater BMI reductions. Gunnarsdottir, et al. [55] assessed importance, confidence and readiness with a questionnaire for parents. The outcome measures were treatment completion and weight change at three time points. Results were mixed: Confidence (but not readiness and importance) was lower in parents who dropped out than in completers. Confidence also predicted weight change at early response and at treatment end, but not at follow-up. Braet, et al. [61] asked the study team to categorize parental and children’s motivation as l*ow*, *moderate* or *strong*. They found that parents of children who completed the treatment were significantly more motivated, but there was no difference in the children's motivation. Detailed information can be seen in Table 4.

In contrast to this, there were three studies [53, 54, 63] showing no predictive power of motivation for the outcome variables. For example, Rojo, et al. [63] assessed maternal importance and self-perceived willingness and found no association with attendance (used as an operationalization of adherence). This leads to the conclusion that results are diverse. There are some studies showing associations between motivation and outcomes and some studies which could not find that motivation predicted outcomes. Different outcomes, assessments of motivation and target persons additionally complicate the comparability of results which leads to no clear conclusion.

### Risk of bias

The overall study quality of included studies was weak with only one study being rated *strong* [50] and two studies being rated *moderate* [36, 59]. The category *withdrawal* often received a *weak* rating indicating high dropout rates in many studies which is a well-known problem in weight reduction literature [71]. Overall, this rating shows that it is challenging to perform good studies with families suffering from OB/OW. The detailed ratings can be found in Table 5.

**Table 5:** Results of the EPHPP rating (3: weak rating, 2: moderate rating, 1: strong rating)

	Selection bias	Study design	Confounders	Blinding	Data collection	Withdrawal	Total rating
Accurso et al., [64]	3	2	3	3	3	3	3
Braet et al., [61]	3	2	3	3	1	3	3
Boff et al., [36]	2	1	1	2	1	3	2
Da Silva et al., [52]	3	2	3	3	1	3	3
Dhuper et al., [53]	2	2	3	3	3	N/A	3
Delamater et al., [37]	2	2	3	3	1	3	3
Ehrmann et al., [54]	3	2	3	3	3	3	3
Fenner et al., [65]	2	3	3	3	1	3	3
Fonvig et al., [38]	2	2	3	3	3	3	3
Gunnarsdottir et al., [55]	2	2	3	3	2	2	3
Gourlan et al., [50]	2	1	1	2	1	1	1
Gourlan et al., [49]	2	2	3	3	1	3	3
Ham et al., [48]	2	1	3	3	1	1	3
Jørgensen et al., [39]	2	2	3	3	2	3	3
Kahana et al., [46]	2	1	3	3	1	3	3
Kotler et al., [40]	3	2	3	3	1	3	3
Lee et al., [41]	2	1	3	3	3	2	3
Luca et al., [42]	2	2	3	3	1	2	3
MacDonell et al., [51]	2	1	3	2	1	3	3
MacDonell et al., [66]	3	1	3	2	1	3	3
Mâsse et al., [58]	3	2	3	3	1	3	3
Naar et al., [59]	3	1	1	2	1	1	2
Reinehr et al., [60]	3	2	3	3	3	3	3
Rodriguez et al., [56]	1	3	3	3	3	N/A	3
Rojo et al., [63]	3	1	1	3	3	3	3
Schiel et al., [45]	2	2	3	3	3	3	3
Segalla et al., [67]	2	1	1	3	1	3	3
Taylor et al., [57]	2	1	3	3	1	N/A	3
Van Allen et al., [62]	3	1	3	3	1	2	3
Van Rinsum et al., [44]	2	2	3	3	1	3	3
Verloigne et al., [47]	3	2	1	3	1	3	3
Watson et al., [43]	2	1	1	3	1	3	3

## Discussion

This systematic review aimed to investigate the role of motivation in weight reduction programs for children and adolescents with OB or OW. We systematically summarized evidence on the theoretical frameworks of motivation used in the studies and the effects of motivation when used as predictor or outcome. After the screening process, 32 studies were included in this review. Risk of bias of these studies was rated with the Quality Assessment Tool for Quantitative Studies by the Effective Public Health Practice Project (EPHPP) [68, 69] leading to mostly weak ratings. Therefore, there may be risk of bias in included studies, but a systematic risk of bias seems unlikely.

Regarding the first research question, we found that the SDT and the TTM are the most commonly used theoretical frameworks, while the questionnaires used vary widely. Based on the TTM, MI is often implemented as a motivation-enhancing therapeutic approach, and readiness rulers are frequently used to measure the level of motivation. The TTM seems to be a good approach to address motivation in weight reduction programs because it uses ambivalence as a central feature which also plays a major role in families participating in weight reduction programs. In contrast, the SDT also seems suitable because its approaches to promote autonomy, competence and social integration can be easily addressed in weight reduction programs for children and adolescents. However, it became also clear that authors based their weight reduction programs and assessments of motivation on very different theoretical frameworks. This leads to an impairment of the comparability of the described outcomes and makes it harder to synthesize the outcomes of the included studies. The diversity also shows that there is disagreement on the construct of motivation and how to operationalize and measure it. A “gold-standard” of measuring motivation, especially in the field of OB and OW in children and adolescents, is missing and strongly needed.

The second research question refers to the change of motivation as an outcome variable of weight reduction programs. We found 18 studies to answer this question. The majority of studies could find higher motivation values after the program than at baseline, indicating an increase. Based on the included evidence, it can be partly confirmed that participation in weight reduction programs can increase motivation. Weight reduction goes along a with a lifelong change in lifestyle and does not end with the end of the weight reduction program. Families need to continue what they have learned which can be challenging. Therefore, a higher level of motivation after the weight reduction program can be helpful to maintain treatment outcomes over the time after the program.

With regard to a link between motivation and weight change there is no clear conclusion possible since results are mixed. An association between weight change and motivation over studies could not be found. This raises the question about the benefits of an increased motivation, given that there is limited association with weight change as the main goal of weight reduction programs. Another criticism about the included studies for this research question, is that all but one study assessed only the children’s or adolescents’ motivation. Therefore, the results are only valid for children’s and adolescents’ motivation, but not for parental motivation. As explained before, especially in younger children the treatment of OB/OW needs to be directed to the whole family since parents act as *agents of change *[19]*.* Therefore, not only the children’s motivation is important, but also parental motivation plays a central role and should be assessed as well. Additionally, there might also be a link between children’s and parental motivation which is of interest.

As the third research question we aimed to find out if motivation can act as a predictor for success in weight reduction programs. The included studies used different outcomes such as weight change at different time points, adherence, dropout or the uptake of a weight reduction program. Additionally, most studies used different subscales or data from parents and children as multiple predictors. Therefore, the data are inconclusive, consisting of studies showing predictive effects of motivation or at least some subscales and studies which showed no effect. A conclusion cannot be drawn. Although the data in our review did not show it, we still assume that baseline motivation plays a role for several outcomes of weight reduction programs. The variety of different variables in the included studies might be a reason why no relation over the studies was found. More homogenous assessments of motivation and outcomes might have led to a clearer result which leads back to the claim of a gold standard for the assessment of motivation. Focusing on dropout as one of the outcome variables here, is important because dropout rates are known to be high in childhood weight reduction programs, ranging between 27–73% [71]. Reasons for dropout are analyzed in many studies, however motivation is barely included as a potential factor. In contrast, the included study of Braet, et al. [61] found three predictors of dropout which were age, psychopathology of the child and motivation of the parent. In a systematic review by Skelton and Beech [71] which summarized dropout reasons from childhood weight reduction programs, motivation is not included. This means that either there is no link between motivation and dropout or this link was not found because it is rarely examined. This association is better analyzed in the literature about weight reduction programs in adulthood, for example in the systematic review by Moroshko, et al. [72]. Some of the studies included in their review assessed self-efficacy and motivation in relation to attrition. Although the results are not clear, they show a trend that lower self-efficacy and lower motivation scores are associated with higher attrition scores. This link might also exist in childhood weight reduction programs as was also shown in some of the included studies in the current review [55, 61].

The results of the current review are important since on the one hand they show that motivation plays an important role in weight reduction programs for children and adolescents, but on the other hand we need more research using more similar theoretical frameworks and assessments of motivation to make results better comparable.

Furthermore, another criticism about the literature and research on childhood weight reduction is that only a few studies assessed motivation at all. During the screening of the results it became clear that many weight reduction programs do not assess the level of motivation of the participating children or parents. Another huge amount of studies did not include the aspect of motivation at all, i.e., neither assessing it, nor using motivational approaches in the treatment although this is recommended in the guidelines. Additionally, for future research, not only a pre and post-assessment of motivation would be desirable, but also an assessment of the progress of motivation. In doing so, clinicians could realize when families face barriers and could intervene at the right time point to prevent dropout or failures. According to its study protocol [73], the STARKIDS study shows a promising approach thereof. It focuses on motivation, by assessing it multiple times, using MI and offering options for motivational barriers.

### Clinical perspectives

We know that there is high prevalence of OB and OW in children and adolescents, but only a subgroup seeks treatment. Reasons are capacity of treatment options and that weight reduction programs do not meet families’ needs [74], but also that there are parents who do not perceive their children as affected by OW/OB [75] and therefore do not seek treatment. The two included studies [56, 57] which analyzed the influence of motivation on the uptake of a weight reduction program found that families who are better aware of the OB/OW status of their child, were more likely to take up the offered weight reduction program. Other factors which motivate families to engage in weight reduction programs are found in a systematic review by Silva, et al. [33]. They found that adolescents most often reported better health, esthetic reasons, improvements in self-esteem and avoidance of bullying to be their motivations for weight loss. Parents reported, e.g., concerns about their child and recommendations of their physician as reasons for a desired weight loss [76]. Rojo, et al. [63] found that an initial orientation session could enhance engagement in the initial phase of the treatment which was also identified as a strategy to reduce attrition [77].

### Strengths and limits

To the best of our knowledge, we offer the most recent and comprehensive synthesis on the role of motivation in pediatric weight-loss programs. By including studies with different theoretical frameworks and different applications of motivation, we provide a comprehensive overview on the field. However, due to the heterogeneity of the studies, it is difficult to draw conclusions or to perform a meta-analysis. In addition, most included studies show weak study quality according to the EPHPP rating [69], hence, single study results must be interpreted with caution. Participants younger than five years and older than 15 years are underrepresented in the original research. A large part of the included studies was conducted in Western countries which makes the results difficult to transfer to other countries, especially to less-developed countries.

## Conclusions

Understanding the role of motivation in weight reduction programs for children and adolescents affected with OB and OW is important to be able to use motivational aspects better in future programs. The current review shows that treatment programs for pediatric weight loss can enhance participants’ motivation. This can be helpful for children and adolescents to maintain treatment outcomes. Whether motivation can predict treatment success remains unclear; the results of the included studies are too heterogeneous. If future studies could show that baseline motivation predicts therapy outcomes, clinicians could target this time point and try to enhance motivation before entering the weight reduction program to prevent dropout and reduce failures and ultimately reduce costs. In addition, next to using motivation-enhancing methods, continuously tracking the families’ level of motivation might prevent dropout and provides data on the course of motivation.

### What is already known on this subject?

Overweight and obesity in children and adolescents are increasing worldwide and treatment options are rare. Current treatment guidelines recommend using motivational aspects in treatment. Motivation plays a major role in changing one’s behavior—yet its role in the treatment of childhood obesity is not clear yet.

### What this study adds?

Weight reduction programs for children and adolescents use different theoretical frameworks of motivation, most commonly used are the Transtheoretical model and the Self-Determination theory. Motivation of children and adolescents who participate in weight reduction programs was higher after the program than before, which means that most of the included weight reduction programs could increase motivation. The increase of motivation over the course of a weight reduction program can help to maintain healthy behaviors after the end of treatment which can prevent relapses. To assess the predictive power of baseline motivation, further research is needed.

## Data Availability

No datasets were generated or analysed during the current study.
